# Physicians’ perspectives on using a patient decision aid in female stress urinary incontinence

**DOI:** 10.1007/s00192-022-05344-w

**Published:** 2022-09-12

**Authors:** Maria B. E. Gerritse, Carlijn F. A. Smeets, John P. F. A. Heesakkers, Antoine L. M. Lagro-Janssen, C. Huub van der Vaart, Marieke de Vries, Kirsten B. Kluivers

**Affiliations:** 1 Department of Gynecology, Radboud University Medical Center, Geert Grooteplein Zuid 10, Postbus 9101, 6500 HB Nijmegen, The Netherlands; 2 Department of Obstetrics and Gynecology, Gelderse Vallei Hospital, Ede, The Netherlands; 3 Department of Urology, Maastricht University Medical Centre, Maastricht, The Netherlands; 4 Department of Primary and Community Care, Radboud University Medical Center, Nijmegen, The Netherlands; 5 Department of Gynecology, University Medical Centre Utrecht, Utrecht, The Netherlands; 6 Institute for Computing and Information Sciences, Radboud University, Nijmegen, The Netherlands

**Keywords:** Barriers, Facilitators, Patient decision aid, Physician, Shared decision making, Stress urinary incontinence

## Abstract

**Introduction and hypothesis:**

A treatment choice for female stress urinary incontinence (SUI) is preference sensitive for both patients and physicians. Multiple treatment options are available, with none being superior to any other. The decision-making process can be supported by a patient decision aid (PDA). We aimed to assess physicians’ perceptions concerning the use of a PDA.

**Methods:**

In a mixed methods study, urologists, gynecologists and general practitioners in the Netherlands were asked to fill out a web-based questionnaire. Questions were based on the Tailored Implementation for Chronic Diseases checklist using the following domains: guideline factors, individual health professional factors, professional interactions, incentives and resources, and capacity for organizational change. Participants were asked to grade statements using a five-point Likert scale and to answer open questions on facilitators of and barriers to implementation of a PDA. Outcomes of statement rating were quantitatively analyzed and thematic analysis was performed on the outcomes regarding facilitators and barriers.

**Results:**

The response rate was 11%, with a total of 120 participants completing the questionnaire. Ninety-two of the physicians (77%) would use a PDA in female SUI. Evidence-based and unbiased content, the ability to support shared decision making, and patient empowerment are identified as main facilitators. Barriers are the expected prolonged time investment and the possible difficulty using the PDA in less health-literate patient populations.

**Conclusions:**

The majority of physicians would use a PDA for female SUI. We identified facilitators and barriers that can be used when developing and implementing such a PDA.

## Introduction

Many women experience stress urinary incontinence (SUI) as a bothersome symptom with a negative impact on their quality of life [1]. Pelvic floor muscle therapy (PFMT) and synthetic midurethral sling (MUS) surgery are the most common nonsurgical and surgical treatments [2–4]. With PFMT, 32% of women with moderate to severe SUI according to the Sandvik index experience satisfactory reduction of SUI and 16% are subjectively cured [5, 6]. MUS surgery has higher success rates. with 62% to 98% of women being subjectively cured [7]. However, PFMT bears no risk of serious adverse events. Placement of an MUS can cause complications, including overactive bladder complaints, obstructive voiding, and mesh-associated problems such as pain and erosion [7]. Health-related quality of life improves with both treatment options [8, 9] and is higher after MUS surgery [10].

Dutch guidelines recommend advising PFMT and MUS surgery as the primary treatment options in women with moderate to severe SUI [2, 3]. Therefore, choosing a treatment option for this level of SUI is a preference-based decision.

Shared decision making (SDM) is the process in which patients make a treatment decision together with their physician. Three stages can be identified in SDM: patients must be made aware that there is a choice to be made, the different options are discussed, and finally patient and physician make a decision together [11]. The resulting decision is thus based on the available options and also on the patients’ own values regarding their likely benefits and harms.

A patient decision aid (PDA) is a tool that can be used to facilitate SDM [12–14]. It provides information on the medical condition and the various treatment options. Outcomes, such as success rates and possible complications, can be displayed next to each other in an option grid. Harms and benefits are compared. Value clarification exercises can be added to help patients identify their own desired outcomes and level of risk tolerance associated with the treatment [13]. A PDA serves as an addition to counseling of treatment options by the health care professional and without giving specific advice. Use of a PDA leads to better informed patients, less decisional conflict, and less decisional regret [14]. Also, it reduces use of unnecessary tests and elective procedures by supporting SDM [14].

To enhance implementation of a PDA, it is important to identify and take into account patients’ as well as physicians’ perspectives on content and usability [15].

The aim of this study was to identify physicians’ perspectives on factors that can facilitate or obstruct use of a PDA to aid SDM in female SUI.

## Materials and methods

This study is a mixed methods, cross-sectional study using self-reported, online questionnaires and is part of a research project to develop a PDA for women facing a treatment decision for SUI. To our knowledge, this was the first assessment of this specific topic in the Netherlands.

The questionnaires were developed by a research group consisting of three urogynecologists (MG, KK, CvdV), an urologist (JH), a general practitioner (ALJ), and a scientific researcher in the field of gynecology with an affinity for SDM. We developed two separate questionnaires, one for general practitioners (GPs) and one for gynecologists and urologists (medical specialists). In the invitation we included residents—physicians in training for gynecology and urology.

The first part of the questionnaire consisted of questions on physicians’ characteristics and was slightly different for the two groups. Physicians were asked about their daily practice in relation to patients with female SUI, in addition to questions on age, sex, years of practice, and prevalence of SUI in their practice.

The second part of the questionnaire was identical for both groups. This part consisted of 27 statements and 4 open questions that were based on the Tailored Implementation for Chronic Diseases (TICD) checklist [16]. The TICD checklist originates from a systematic review of frameworks and classification of factors that can enable or prevent improvements in health care professional practice. It can be used in practice to aid in designing implementation interventions, such as a PDA.

The research group used the nominal group technique (NGT) to determine which domains of the checklist to use and to achieve consensus on the content of the questionnaires [17]. The NGT is a consensus group method, using expert opinions to reach agreement when evidence is lacking. After preparations, the research group discussed the design and content of the questionnaire in two face-to-face meetings, which resulted in the final version of the questionnaires. Domains of the TICD checklist used were: guideline factors, individual health professional factors, professional interactions, incentives and resources, and capacity for organizational change.

Respondents were asked to fill in their level of agreement on given statements, ranking on a five-point Likert scale from fully agree, agree, no opinion, disagree, to completely disagree. All respondents were asked about their personal opinions on the use of a PDA as a tool to support SDM with regard to content, design, and accessibility.

The online questionnaire was created with the use of SurveyMonkey, an online cloud-based software tool for the creation and distribution of questionnaires.

Data were collected anonymously. We did not apply for approval by an Institutional Review Board, considering that the subjects were physicians; the results were collected anonymously and questions were not obtrusive.

All 275 members of the pelvic floor disorder group of the Dutch Society of Obstetrics and Gynecology as well as all 480 members of the Dutch Society of Urology received an e-mail with an invitation including a link to the questionnaire between November 2016 and April 2017. After 1 month, the (resident) gynecologists received a second e-mail with a reminder. In addition, GPs were approached to participate in the study. We adapted a pragmatic recruitment strategy to reach as many GPs as possible during the inclusion time window, consisting of an invitational e-mail to all 47 GPs with extra training in urogynecology and advertising for the study on regional GP websites. The GPs who were approached directly received a reminder by e-mail 1 month later. Respondents’ characteristics and agreement with statements based on the TCID list were described. For exploratory reasons, we performed a statistical analysis on the levels of agreement of the 27 statements using a Mann–Whitney *U* test to uncover if there were differences in outcomes between gynecologists/urologists and GPs. Two-sided significance levels were used to evaluate the *p* values resulting from the questionnaire and a *p* value of 0.05 was used as cut-off for statistical significance.

We performed a document analysis of the answers in the open questions section. Facilitators of and barriers to the future use of a PDA were identified and grouped into themes. Participants’ quotes were used to illustrate the themes.

## Results

**Fig. 1:**
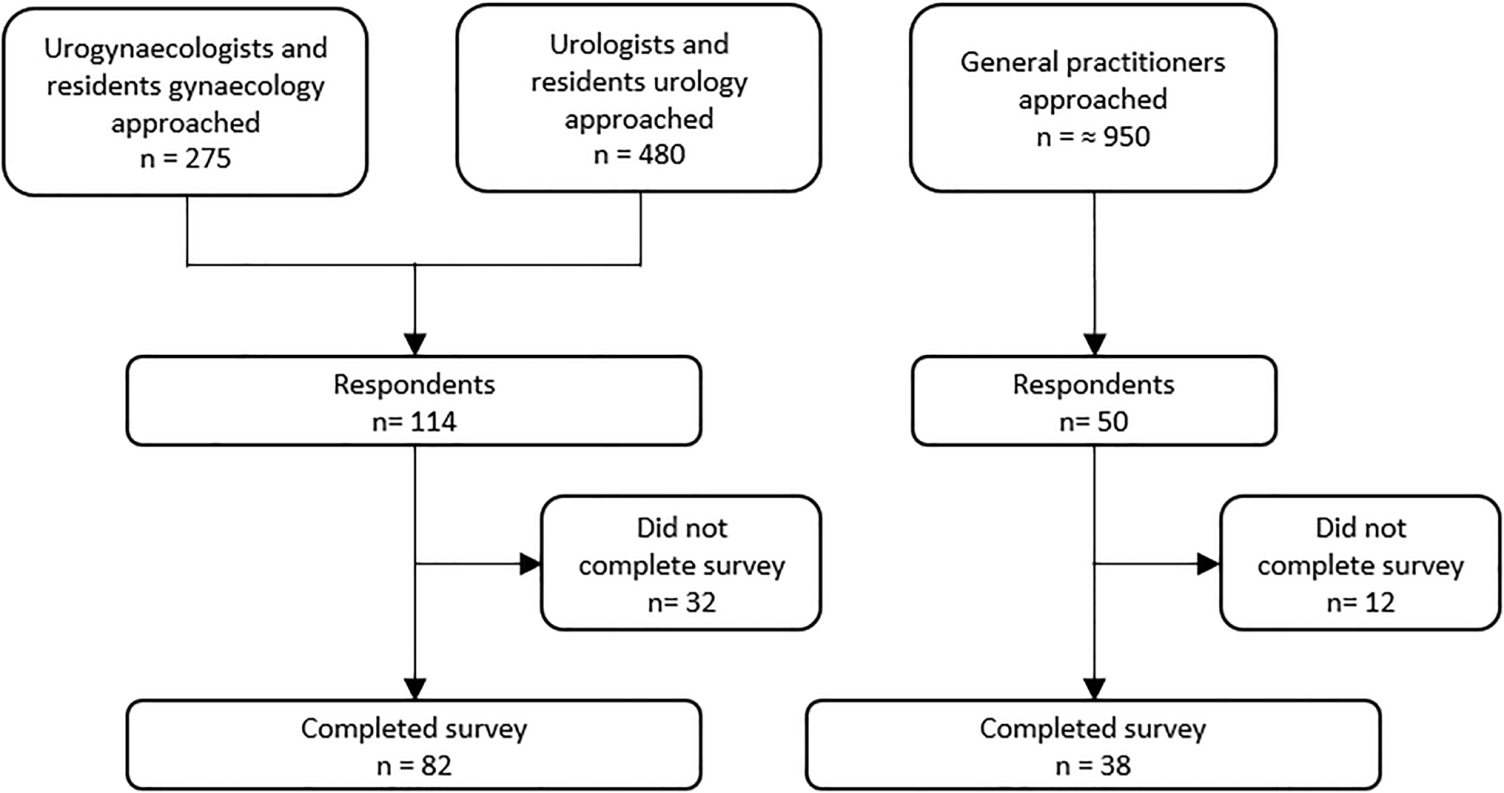
Flow diagram of the response rates of medical specialists and general practitioners: numbers of physicians approached to participate in the study and response rates

Of the (resident) gynecologists and urologists who received an online invitation to participate in the study, 82 medical specialists (11%) completed the mandatory closed answering section. GPs trained in urogynecology were approached directly by e-mail; others had the opportunity to read the advertisement of the study on their regional GP website. The estimated exposure was 950 GPs. Thirty-eight GPs completed the questionnaire. Of this group, 5 GPs had a special interest in urogynecology. The total estimated GP response rate was therefore 4% (Fig. 1).

The female/male ratio in medical specialists was 55%/45%, GPs were more often women, with a female/male ratio of 63%/37%. The mean age in medical specialists was 47 years (range 31–64 years); 7% of the participants were residents. The mean age in GPs was higher at 51 years (range 30–63); there were no participating residents. Tables 1 and 2 list the personal and professional characteristics of the respondents.

**Table 1:** Gynecologists and urologists

Demographic characteristics of respondents (*n*=82)	*n* (%)
Sex	
Female	45 (55)
Male	37 (45)
Age, years	
Mean	47
<40	23 (29)
40–49	28 (35)
≥50	31 (39)
Time practicing as medical specialist, years	
<15	49 (60)
≥15	27 (33)
Resident	6 (7)
Type of practice	
General hospital	70 (85)
Academic hospital	13 (16)
Private clinic	2 (2)
Specialty	
Gynecologist	47 (57)
Urologist	29 (35)
Resident gynecology	1 (1)
Resident urology	4 (5)
Researcher	1 (1)
Estimated new patients with SUI seen by specialism every month	
Average, *n*	17
Estimated MUS placed every month	
Average per practice, *n*	5
Average per specialist, *n*	2

*SUI* stress urinary incontinence, *MUS* midurethral sling, *resident* physician in training for gynecologist or urologist

**Table 2:** General practitioners

Demographic characteristic of respondents (*n*=38)	*n* (%)
Sex	
Female	24 (63)
Male	14 (37)
Age, years	
Average	51
<40	6 (16)
40–49	9 (24)
≥50	23 (61)
Time practicing as general practitioner, years	
<15	9 (24)
≥15	29 (76)
Trained in urogynecology	
Yes	5 (13)
No	33 (87)
Type of practice	
Solo practice	3 (8)
Health center, 2–5 general practitioners	28 (74)
Health center, >5 general practitioners	7 (18)
Area	
Rural	19 (50)
Urban	19 (50)
Estimated new patients with predominant SUI seen every year	
Average, *n*	11
Estimated patients treated by general practitioner	
Average, %	42
Estimated patients sent for referral to pelvic floor muscle therapist as primary treatment	
Average, %	42
Estimated patients sent for referral to gynecologist or urologist	
Average, %	15

*SUI* stress urinary incontinence

All physicians felt that patients should be involved when making treatment decisions and that patients make better decisions when properly informed. Both groups, specialists and GPs, valued the guidance and advice offered by a pelvic floor physiotherapist for women with SUI.

Both groups found themselves to be the most qualified type of physician compared with each other to provide information on SUI and to counsel treatment options. In medical specialists this was 84% in the case of informing and 94% in the case of counseling options for treatment; in GPs these were 79% and 76% respectively.

Gynecologists and urologists were more likely to counsel placement of a MUS as a primary treatment option (93%) or to (advise to) perform surgery without prior PFMT (39%), compared with GPs (68% and 5% respectively). Results of the level of agreement on statements for each group and between the two groups are displayed in Table 3.

**Table 3:** Mean score and agreement between gynecologists/urologists and general practitioners

	Mean score (SD)		Agreement level
	GYN/URO	GP	*p* value
Preferable practitioner giving information about SUI to the patient			
General practitioner	3.52 (±0.88)	2.26 (±0.79)	<0.001
Pelvic floor muscle therapist	3.07 (±0.98)	2.26 (±0.79)	<0.001
Gynecologist or urologist	2.02 (±0.79)	3.34 (±1.02)	<0.001
Preferable practitioner discussing treatment options for SUI			
General practitioner	3.74 (±0.86)	2.29 (±0.73)	<0.001
Pelvic floor muscle therapist	3.65 (±0.85)	2.92 (±0.94)	<0.001
Gynecologist or urologist	1.76 (±0.69)	2.92 (±1.02)	<0.001
Treatment options			
Patients should complete PFMT before considering MUS surgery	2.82 (±1.12)	1.79 (±0.78)	<0.001
Referral to pelvic floor muscle therapist for information and advice on treatment options	2.39 (±0.91)	2.05 (±0.87)	0.044
MUS surgery should be discussed in patients with moderate to severe SUI	1.88 (±0.74)	2.42 (±0.89)	<0.001
Patients must be involved when making treatment decisions	1.32 (±0.47)	1.47 (±0.51)	0.099
Informed patients can make a better treatment decision	1.34 (±0.50)	1.42 (±0.55)	0.463
Patient decision aid			
Should be based on scientific research	1.44 (±0.52)	1.53 (±0.60)	0.520
Should align with urinary incontinence guidelines for specialists or GPs	1.57 (±0.65)	1.71 (±0.69)	0.302
Will be used more often when supported by own scientific organization or representatives thereof	1.70 (0.56)	1.50 (±0.56)	0.073
Use will reduce the time needed for consultation	2.96 (±1.07)	2.82 (±1.06)	0.453
No objection if use of a PDA takes up extra consultation time	3.00 (±1.05)	2.63 (±1.00)	0.073
Will stimulate shared decision making	1.98 (±0.77)	1.92 (±0.54)	0.996
Advise patients to use PDA when it includes information on PFMT and MUS surgery	2.09 (±0.89)	1.97 (±0.72)	0.729

Likert scale: 1 = fully agree, 2 = agree, 3 = no opinion/indifferent, 4 = disagree, 5 = fully disagree

Statistics used: Mann–Whitney *U* test

*SUI* stress urinary incontinence, *PDA* patient decision aid, *PFMT* pelvic floor muscle therapy, *MUS* midurethral sling, *GP* general practitioner

Of the participating physicians, 77% would use a PDA for female SUI when available. Several facilitating factors for the use of a PDA were identified (Tables 3, 4). The content should be based on scientific research (97% of physicians agree) and guidelines (90% agree). Ninety-six per cent of physicians would use it more willingly if a PDA were supported by their scientific organization.

**Table 4:** Facilitators for use of a patient decision aid

Theme	Respondent	Quote
Evidence-based, unbiased information	SP 6	Objective information from a different source
	SP 11	Good-quality information, it helps to make a well-supported treatment decision
	SP 27	I hope it will give insight into the successful treatments, with the use of an explanatory figure, for example
Uniformity in information provisioning and counseling	SP 31	Uniformity between different health care professionals. Unambiguous information. To show patients how they can make their choice
	SP 33	Clear, standardized way of information provision
Support of shared decision making	SP 52	Support for both patient and physician to make a well-substantiated choice
	SP 60	To have the patient make a better informed, good treatment decision that fits her values
	GP 20	Making a treatment decision together with the patient and discussing all the important subjects
Patient empowerment	SP 22	If it helps the patient to better weigh the advantages and disadvantages
	SP 25	I very much believe in a patient’s right to self-determination when she is properly informed
Patient preparation for the consultation	GP 8	It is useful to help somebody to think it over by themselves
	GP 19	Preparation of the consultation will take less time. Knowledge in the patient, she can think about it
	GP 31	A patient can judge for herself already what is applicable for her or not
Saving time during consultation	GP 33	Better considerations, time saver

*GP* general practitioner, *SP* medical specialist

Physicians felt it hard to predict if use of a PDA would result in a longer or shorter duration of patient–physician consultation: 36% expected a shorter duration, 33% expected it to take more time. Forty-three per cent would still use a PDA in the case of a prolonged consultation time; for 37%, this would be an objection to using a PDA.

### Facilitators and barriers

We identified six themes of facilitators in the open answer section, with quotes from 104 participants: evidence-based and unbiased information, uniformity in information provisioning and counseling, support of SDM, empowerment of the patient, patient preparation for consultation, and saving time during consultation (Table 4).

The majority of physicians recognized the need for SDM in the process of choosing a treatment option for female SUI and the support a PDA can provide in this process: “Support for both patient and physician to make a well-substantiated choice” (SP 52).

Evidence-based and unbiased information was also considered important, as was already seen in the statement part of the questionnaire. Physicians expected the information on different treatments to be more evidence based. Also, hope was expressed that by using different forms of explanations such as the consultation itself and several forms of information displayed both visually and verbally on the PDA, information will be easier to understand for different types of patients: “I hope it will give an insight into successful treatments, with the use of an explanatory figure, for example” (SP 27).

Associated with this is the importance of uniformity in information provision and counseling. A medical specialist (SP 31) wrote as a possible facilitator: “Uniformity between different health care professionals. Unambiguous information. To show patients how they can make their choice.”

Use of a PDA was believed to empower patients, as was said: “I very much believe in a patient’s right to self-determination when she is properly informed” (SP 25) and “To give the patient more direction to make a responsible choice” (GP 35). A PDA can help to prepare the patient for the consultation with her physician, as GP 19 said, “Preparation of the consultation will take less time. Knowledge in the patient, she can think about it” (GP19). Finally, an expected reduction in the consultation time was named as a facilitating factor.

Four themes were identified as barriers in the open answer section: time consuming, illiteracy and/or a lack of understanding the Dutch language in patients, biased content with a preference for surgery, and physicians’ doubts about the additional value of a PDA (Table 5). The majority of physicians named fear of a longer duration of the consultation as a barrier. Also, physicians feared that a PDA will be too difficult to comprehend or use by health-illiterate people, women lacking a good understanding of the Dutch language, or those unable to use a computer or the internet. Biased content with a preference for surgery in a PDA would obstruct use.

**Table 5:** Barriers to the use of a patient decision aid

Theme	Respondent	Quote
Time consuming	SP 52	It should not take a lot of time during the consultation
Illiteracy and/ or lack of understanding Dutch language in patients	SP 30	Lack of a good understanding of the Dutch language, illiterate people
	GP 14	Unable to use a computer or the internet
Biased content with a preference for surgery	SP14	If the PDA should question pelvic floor muscle therapy. I find PFMT useful for explanation, lifestyle advice, prevention after surgery, and for advice on the urgency component of the complaints, which is often present
	GP 32	When it is too much directed toward surgery
Physicians’ doubts about the additional value of a PDA	SP 63	Nothing is better than a good conversation between a physician and a patient
	GP 8	Sometimes it is a simple choice

*GP* general practitioner, *SP* medical specialist, *SUI* stress urinary incontinence, *MUS* midurethral sling

## Discussion

### Principal findings

To our knowledge, this is a first assessment of physicians’ perspectives on using a PDA in female stress urinary incontinence. All participating physicians in this study support SDM and feel that patients will make qualitatively better decisions when properly informed. The majority of participants is willing to use a PDA as an addition to counseling treatment options. Reliable, evidence-based, and unbiased content is valued as an important facilitator for the use of a PDA. This was also seen in previous research on the implementation of health care adaptations [16]. Participants think that SDM will be supported by use of a PDA and that patients are better informed and more empowered to participate in the SDM process. The positive effect on SDM and patient empowerment has been shown before in other studies [14, 18, 19].

Possible prolongation of consultation time was seen as the greatest barrier to implementing a PDA. Only 43% of the physicians in our study would still use it if consultation time were to increase. Several other studies mention expected prolongation of consultation time as a physicians’ barrier [14, 18, 20, 21]. The Cochrane review by Stacey et al. showed that the median increase in consultation time is 2.6 min when using a PDA during a consultation [14]. Only 2 out of 7 studies included in the review, on atrial fibrillation and on prenatal counseling, did show an increase in time spent during consultation; all the others did not. Informing physicians about such a low chance of a relevant increase in consultation time may well increase PDA use. Introduction of the PDA before the consultation itself can also reduce the duration of the consultation and has the same positive effects on the SDM process as applying a PDA during the consultation itself [14]. In this case, identification of the patients’ complaints must have taken place before the consultation to send the PDA beforehand.

Health illiteracy, poor understanding of language, problems with reading or use of the internet by patients were identified as other important barriers. Health-illiterate patients can benefit even more than literate patients from SDM interventions in terms of increased knowledge, informed choice, participation in decision making, decision self-efficacy, and reduced decisional conflict [22, 23]. However, care and extra attention should be given to tailor interventions to lower literacy needs [22, 24].

In an exploratory analysis we identified differences in counseling treatment options between medical specialists and GPs. Both groups of physicians consider themselves to be best suited to informing and counseling women with SUI. However, gynecologists and urologists are more likely to advise MUS surgery than GPs, although recommendations for counseling SUI treatment options do not differ between the guidelines for GPs and medical specialists in the Netherlands [2, 3]. A possible explanation is that patients in first- and second-line care differ with regard to the severity of SUI complaints and also with regard to their readiness to undergo operative treatment. In addition, it is possible that there is a difference in counseling treatment options between first- and second-line care independent of the severity of complaints. Differences in counseling can lead to several unwanted effects, such as ineffective care, increased costs, and emphasis on physicians’ preferences rather than patients’ preferences [25]. SDM and use of a PDA may reduce the counseling differences by offering uniformity of information [25–27].

### Strengths and limitations

One of the strengths of this study is that we included physicians from all medical specialties that inform and counsel women with SUI in the Netherlands. We reached all Dutch (resident) gynecologists, (resident) urologists, and GPs trained in pelvic floor problems with our study invitation. The response rate of 11% of the medical specialists and 10% of the GPs trained in urogynecology is concordant with earlier research using online surveys with a personal invitation [28]. The total GP response rate is one of the weaknesses of the study: 5% based on an estimated number of 950 GPs reached with the study invitation, whereas a total of 12,127 GPs were practicing in the Netherlands in 2014, the year in which this research commenced [29]. The GP response rate is difficult to interpret, because most of these participants only learned about the questionnaire when visiting a website for other purposes and could not be reached personally owing to privacy issues.

### Implications for the future

The identified facilitators for and barriers to PDA use in female SUI should be taken into account when developing and implementing such a tool. Extra attention needs to be given to educating physicians on the limited time investment with use and on the advantages of PDA use for health-illiterate patients when the tool is tailored to their needs. Use of a PDA may decrease differences in counseling treatment options in female SUI.

## Conclusions

Most physicians are willing to use a PDA in female SUI. In a PDA, physicians most value evidence-based, unbiased content, patient empowerment, and support of SDM. Important barriers to PDA use are an expected increase in consultation time and difficulty using in the case of health illiteracy. Differences in counseling SUI treatment options exist between primary and secondary care physicians.

## Questionnaires

### Questionnaire for gynecologists and urologists, first part

1. What is your gender?
2. How old are you?
3. What is your function? Options: gynecologist with interest in urogynecology, urogynecologist, urologist with interest in functional urology, functional urologist, urologist without special interest in functional urology, resident gynecology, resident urology, other.
4. How many years have you been working as a medical specialist?
5. In what kind of clinic do you work? Options: academic center, resident training hospital, general hospital, private clinic
6. What is the prevalence of women with complaints of stress urinary incontinence for your own specialty in your clinic per month?
7. How many midurethral slings (MUS) are placed in your clinic by physicians of your own specialty?
8. How many MUS do you place yourself per month?

### Questionnaire for general practitioners (GPs), first part

1. What is your gender?
2. How old are you?
3. What is your function? Options: GP without specialization, GP specialized in urogynecology, other specialization, resident trainer, researcher.
4. For how many years have you been working as a GP?
5. In what kind of practice do you work? Options: solo practice, health center with 2–5 GPs, health center with more than 5 GPs
6. Where is your practice located? Options: city, rural area
7. What is the prevalence of predominant SUI in your practice?
8. What percentage of women do you treat yourself at first?
9. What percentage of women do you refer for primary pelvic floor muscle therapy (PFMT)?
10. What percentage of women do you refer primarily to a gynecologist or urologist?
11. Do you provide information on SUI or refer to another source of information yourself?
12. Do you let your patients fill in a bladder diary?
13. Do you perform a urine check?
14. Do you counsel patients on the treatment choice between PFMT and MUS surgery?

### Questionnaire for all physicians, second part

#### Statements

1. I am aware of the content of the multidisciplinary guideline “Urinary incontinence for 2nd and 3rd line care” (2013, update 2014).
2. I am aware of the content of the GP guideline “Urinary incontinence in women” (2015).
3. I am aware of the most important results for the Dutch study, which randomized between primary MUS surgery and PFMT in moderate to severe female SUI (Labrie et al. [5])
4. The results of the study by Labrie et al. [5] has made me perform primary MUS surgery more frequently (only gynecologists and urologists).
5. The patient information that is available on SUI is reliable.
6. The patient information that is available on SUI is up to date.
7. The patient information that is available on SUI treatment options is reliable.
8. The patient information that is available on SUI treatment options is up to date.
9. I am of the opinion that there are enough information options on SUI for patients (for example, websites, patient leaflets).
10. The general practitioner is the most frequently designated health care professional to provide information on SUI.
11. The pelvic floor therapist is the most frequently designated health care professional to provide information on SUI.
12. The gynecologist and urologist are the most frequently designated health care professionals to provide information on SUI.
13. The general practitioner is the most frequently designated health care professional to counsel on treatment options in SUI.
14. The pelvic floor therapist is the most frequently designated health care professional to counsel on treatment options in SUI.
15. The gynecologist and urologist are the most frequently designated health care professionals to counsel on treatment options in SUI.
16. I feel that SUI patients should first do PFMT before proceeding to MUS surgery.
17. Advice and explanations given by a pelvic floor therapist are reasons for me to refer for PMFT first.
18. I feel that primary MUS surgery should be discussed with women with moderate to severe SUI.
19. I feel that patients should be involved in making a treatment decision on SUI.
20. I feel that patients who are well informed are better able to make a treatment decision.
21. I think that it is important for information in the PDA to be evidence based.
22. I find it important for the PDA to be connected to the guidelines of GPs and medical specialists.
23. I will use the PDA more easily when it is supported by (representatives of) my scientific society.
24. I expect to reduce consultation time when using the PDA.
25. If using the PDA would mean that consultation time increases, I would not object.
26. I expect that use of the PDA will enhance the treatment decision conversation and shared decision making.
27. If there is a PDA aimed at easing the choice between PFMT and MUS surgery, I would recommend this to all my patients.

#### Open questions

1. What topics would you like to see addressed in the PDA?
2. What are the reasons for you using the PDA?
3. What are the reasons for you not using the PDA?
4. The PDA is web based. How should the website be available?
